# Inhibitor of Growth Proteins: Epigenetic Regulators Shaping Neurobiology

**DOI:** 10.3390/biom15020281

**Published:** 2025-02-14

**Authors:** Ziyue Xu, Hongyu Xu, Jichun Shi, Runming Liu, Xiang Li, Sha Liu, Wei Wei

**Affiliations:** 1 Brain Research Center, Zhongnan Hospital of Wuhan University, Wuhan 430071, China; xzy0727@whu.edu.cn (Z.X.); xuhongyu@whu.edu.cn (H.X.); sjc2024@163.com (J.S.); 2023203030035@whu.edu.cn (R.L.); li.xiang@whu.edu.cn (X.L.); 2 Department of Neurosurgery, Zhongnan Hospital of Wuhan University, Wuhan 430071, China; 3 Frontier Science Center for Immunology and Metabolism, Wuhan University, Wuhan 430071, China; 4 Medical Research Institute, Wuhan University, Wuhan 430071, China; 5 Sino-Italian Ascula Brain Science Joint Laboratory, Zhongnan Hospital of Wuhan University, Wuhan 430071, China; 6 Department of General Practice, Zhongnan Hospital of Wuhan University, Wuhan 430071, China

**Keywords:** ING, epigenetic regulation, neurons, genes, molecular pathways, neurodevelopment

## Abstract

The inhibitor of growth (ING) family of proteins is emerging as a pivotal regulator of epigenetic modifications within the nervous system. These proteins are involved in various cellular processes, including apoptosis, cell cycle control, and DNA repair, through interactions with chromatin-modifying complexes. Recent studies underscore the dual role of ING proteins in both tumor suppression and neuronal differentiation, development, and neuroprotection. This review summarizes the epigenetic functions of ING proteins in neurobiology, with a focus on their involvement in neural development and their relevance to neuro-oncological diseases. We explore the mechanisms by which ING proteins influence chromatin state and gene expression, highlighting their interactions with histone acetyltransferases, deacetylases, histone methyltransferases, DNA modification enzymes, and non-coding RNAs. A deeper understanding of the role of ING proteins in epigenetic regulation in the nervous system may pave the way for novel therapeutic strategies targeting neurological disorders.

## 1. Introduction

The inhibitor of growth (ING) family members are key regulators in the field of epigenetic regulation. The first member, *ING1*, was identified in 1996 as a tumor suppressor gene [1]. Since then, the *ING* family has expanded to include five isoforms (*ING1* to *ING5*), all of which have been shown to play a range of roles in a variety of biological processes, including cell cycle transition, cellular senescence, DNA damage repair, apoptosis, angiogenesis, and chromatin structure modulation [2,3]. Recent studies have shown that the functions of ING proteins extend beyond tumor suppression, particularly in the central nervous system, where they serve as crucial epigenetic regulators [4].

ING proteins primarily regulate the chromatin landscape through interactions with various epigenetic complexes, thereby influencing gene expression [5]. For instance, ING proteins are connected to the nucleosome remodeling and deacetylation complex (NuRD), which influences gene transcription and expression [6]. Additionally, ING proteins interact with histone acetyltransferases (HATs) and histone deacetylases (HDACs), which modulate chromatin accessibility [7,8] and, consequently, gene expression. The epigenetic mechanisms are essential for neuronal growth and development.

Epigenetics refers to the study of gene regulation mechanisms that do not alter the DNA sequence but instead regulate gene expression through processes such as histone modification, DNA/RNA methylation, and the activity of non-coding RNAs [9,10]. These regulations are crucial not only for cell differentiation, development, and physiological homeostasis, but also for the pathogenesis of various diseases, including cancer, brain injury, and neurological and metabolic disorders [11,12]. In the context of neuroscience, epigenetic changes have a profound impact on gene expression, influencing neuronal function, synaptic plasticity, and neural signaling [13]. Specifically, in processes such as learning and memory, epigenetic modifications enhance or suppress neural activity by regulating synaptic plasticity and neural circuits [14].

Although the roles of ING proteins in neuroscience are beginning to be understood, their specific epigenetic regulatory mechanisms and interactions with other molecules remain areas of active investigation. Further analysis of the functions and mechanisms of ING proteins in neuronal morphogenesis, neurobiology, disease pathogenesis, and potential therapies will deepen our understanding of the nervous system and related diseases. This could pave new avenues toward the common goal of treating neurological disorders and neurodevelopmental disorders.

In summary, the role of ING proteins as epigenetic factors in neuroscience is noteworthy and warrants further exploration. In this review, we explain their roles in neurobiology based on epigenetic pathways such as histone modifications, DNA/RNA modifications, and ncRNA regulation.

## 2. Structural Domains and Functional Roles of ING Proteins

**Figure 1:**
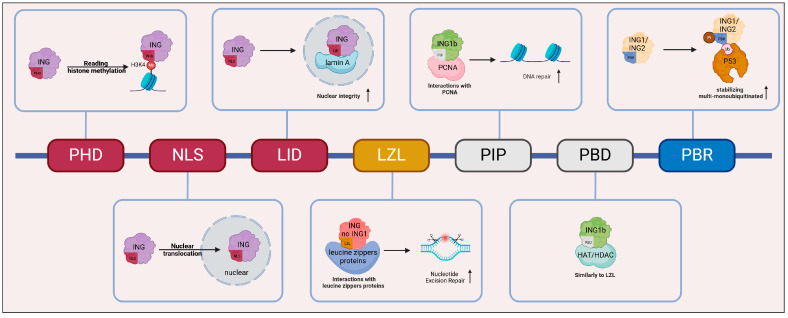
Structure of ING protein. The red regions (PHD, NLS, and LID) represent domains common to all ING proteins, the yellow region (LZL) represents a structure shared by all ING proteins except ING1, the gray regions (PIP and PBD) represent domains specific to ING1b, and the blue region (PBR) represents a domain specific to ING1 and ING2. Each domain-linked block represents a functional diagram. Abbreviations: PHD, plant homeodomain; NLS, nuclear localization signal; LID, lamin-interacting domain; LZL, leucine zipper-like; PIP, PCNA-interacting protein; PBD, partial bromodomain; PBR, polybasic region; PI, phospholipid; Ub, ubiquitin. This graph was created with BioRender.com.

ING proteins are multi-domain, highly conserved proteins that exhibit significant homology across their family members [15]. While the N-terminal domains of each family member are distinct, they share similar overall structural features. All ING proteins contain three conserved regions: the lamin-interacting domain (LID), the nuclear localization signal (NLS), and the plant homeodomain (PHD) motif (Figure 1). The NLS domain directs ING proteins to the nucleus by binding to nucleolar proteins, thereby facilitating their functional roles within the nuclear compartment. For example, under stress conditions, targeting ING1 to the nucleus promotes apoptosis [16]. The highly conserved PHD motif, consisting of 50 amino acids, activates gene transcription by binding to the histone markers H3K4me3 and H3K4me2 [17,18]. The LID is essential for interacting with lamin A and could maintain nuclear morphology, at least in the case of ING1 [19]. This interaction is important for the structural integrity of the nucleus. In addition to these common features, ING proteins, with the exception of ING1, contain a unique leucine zipper-like (LZL) motif. This motif facilitates interactions with proteins that possess leucine zippers, such as the oncoproteins Jun, Fos, and Myc, which are functionally involved in the regulation of oncogene expression [20]. Furthermore, it is essential for ING2-mediated nucleotide excision repair and apoptosis [21].

ING1b, a splice variant of ING1, contains a distinct PCNA-interacting protein (PIP) motif (Figure 1) that specifically binds to proliferating cell nuclear antigen (PCNA). By promoting PCNA monoubiquitination and facilitating chromatin relaxation, ING1b enhances the repair of oxidative DNA damage, thereby maintaining genomic stability and preventing oxidative stress-induced apoptosis [22,23]. ING1b also contains a partial bromodomain (PBD), whose function remains unclear, but it may potentially act similarly to the LZL motif by interacting with HATs and HDACs [24]. Another unique feature of ING1 and ING2 is the polybasic region (PBR). This motif enables interaction with bioactive signaling phospholipids (PIs) and ubiquitin (Ub), with the latter potentially stabilizing multi-monoubiquitinated p53 [25]. This interaction could be pivotal for the regulation of p53-mediated cellular responses.

## 3. Epigenetic Regulatory Role of ING Proteins

Epigenetic mechanisms are pivotal in the development and functional maintenance of the nervous system, and their dysregulation is strongly associated with the onset of various neurological disorders. For instance, modifications in DNA methylation, histone modifications, and non-coding RNA (ncRNA) regulation can profoundly affect neuronal function, contributing to conditions such as neurodevelopmental and neurodegenerative diseases. One example is the interaction between PROSER1 and Ten-Eleven Translocation (TET) enzymes, specifically in the context of mouse embryonic stem cells (mESCs). PROSER1 stabilizes the chromatin-bound TET-OGT-PROSER1-DBHS (TOPD) complexes, which regulate DNA demethylation and developmental gene expression. Furthermore, PROSER1 sequesters TET enzymes, preventing excessive demethylation and the subsequent derepression of transposable elements, which can lead to genomic instability. This interaction has been linked to neurodevelopmental disorders, as mutations in PROSER1 lead to developmental abnormalities, including craniofacial malformations, hypotonia, and sensorineural hearing loss in humans, mirroring defects observed in the knockout mouse model of PROSER1 [26]. Similarly, other studies have highlighted significant alterations in the DNA methylation patterns within key gene regions, which may be critical for the onset of neurodevelopmental disorders and neurodegenerative diseases [27,28,29].

Histone acetylation, another critical epigenetic modification, also plays a major role in neurological disorders. Imbalances in histone acetylation have been shown to significantly affect gene expression and neuronal function. For example, increasing histone acetylation has been shown to improve sociability and restore learning and memory in KAT6B-haploinsufficient mice [30]. The dysregulation of histone acetylation may also affect the progression of Parkinson’s disease (PD), potentially by altering the survival and synaptic plasticity of dopaminergic neurons [31,32]. Moreover, the dysregulated expression of ncRNAs, including miRNAs and lncRNAs, has been implicated in various psychiatric disorders, such as depression and autism spectrum disorders (ASDs). These ncRNAs mediate the long-term effects of environmental factors on the nervous system by regulating inter-neuronal signaling, synaptic function, and neuroplasticity [33,34]. ING proteins, due to their unique structural domains, have been identified as key players in the regulation of histone modifications. Recent studies have also indicated that ING proteins are involved in the modulation of ncRNAs [35,36]. Collectively, these findings underscore the role of ING proteins in the regulation of neurological disorders through epigenetic mechanisms. Subsequently, we provide a structured discussion on the various epigenetic regulatory mechanisms of ING proteins.

### 3.1. Regulation of Gene Expression by ING Proteins Through Histone Acetylation

Histone acetylation is a fundamental mechanism of epigenetic regulation that plays a crucial role in gene expression by altering the structure of chromatin. This process involves the addition of acetyl groups to specific amino acid residues on histones, primarily lysines [37]. The addition of acetylation reduces the electrostatic interactions between histones and DNA, which in turn relaxes the chromatin structure. This relaxation allows for easier access to the DNA by transcription factors and other regulatory proteins, thereby facilitating gene expression [38]. The N-terminal domains of ING proteins interact with histone-modifying enzymes, including histone acetyltransferases (HATs) and histone deacetylases (HDACs), which regulate the balance between histone acetylation and deacetylation—a process essential for maintaining proper epigenetic regulation [39]. By engaging with these enzymes, ING proteins act as key epigenetic regulators that modulate histone acetylation, thereby influencing chromatin accessibility and gene expression [40].

**Figure 2:**
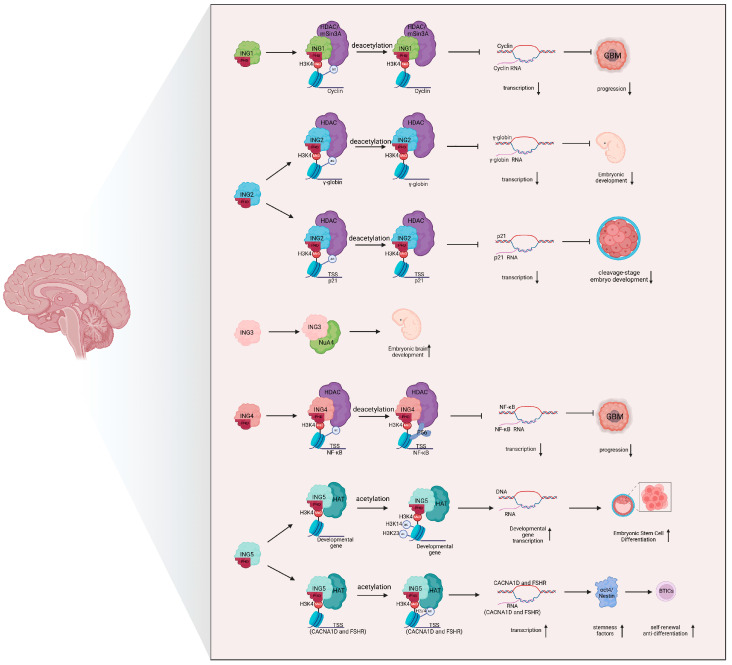
ING proteins and histone acetylation and methylation. Schematic representation of the regulatory mechanisms of ING proteins in the nervous system through interactions with histone acetylation and histone methylation. Abbreviations: PHD, plant homeodomain; HDAC, histone deacetylase; GBM, glioblastoma; NuA4, nucleosome acetyltransferase of histone H4; TSS, transcription start site; HAT, histone acetyltransferase; CACNA1D, calcium voltage-gated channel subunit alpha1 D; FSHR, follicle-stimulating hormone receptor; Oct4, organic cation/carnitine transporter 4; BTICs, Brain-Tumor-Initiating Cells. This graph was created with BioRender.com.

For instance, ING3 is an essential component of the NuA4 histone acetyltransferase complex. It plays a pivotal role in regulating histone acetylation during embryonic development, particularly in the prenatal brain, where proper gene expression is crucial for neurogenesis [41]. By contrast, ING5, which is highly expressed in stem cells, forms a complex with HATs that is vital for maintaining and differentiating neural cells (Figure 2) [42]. Specifically, the ING5-HAT complex targets gene promoters marked by H3K4Me3 in glioblastoma, particularly in Brain-Tumor-Initiating Cells (BTICs). The PHD domain of ING5 aids in the recruitment of HAT complexes, including monocytic leukemia zinc finger protein (MOZ), MOZ-related factor (MORF), and HBO1, to these regions, facilitating histone acetylation. This process activates genes involved in calcium signaling and follicle-stimulating hormone (FSH) pathways, such as CACNA1D and FSHR, and promotes the expression of stemness factors like OCT4 and Nestin. Consequently, the self-renewal and anti-differentiation properties of BTICs are enhanced, supporting glioblastoma progression [43,44].

On the other hand, ING1 exerts an inhibitory role in histone acetylation. It interacts with the mSin3A/HDAC complex to deacetylate histones, which reduces the acetylation levels of histone lysines. This suppression of histone acetylation leads to the downregulation of cell cycle regulators like *Cyclin B1* and *Cyclin D1*, thereby inhibiting the initiation and progression of glioblastomas [6,40,45]. ING4, although expressed at lower levels in gliomas, plays a similar suppressive role in glioma progression. It recognizes H3K4me3 near the *NF-κB* promoter and recruits the HDAC complex to this region, thereby reducing acetylation at the *NF-κB* promoter. This inhibition prevents the activation of *NF-κB*, a key transcription factor involved in inflammation and cell survival, thus impeding tumor growth [46,47,48].

In summary, ING proteins regulate the acetylation status of histones through their interactions with HATs and HDACs. They can either promote gene expression through their interactions with HATs or inhibit gene expression through their interactions with HDACs, thus playing a crucial role in neuronal survival and development.

### 3.2. Regulation of Gene Expression by ING Proteins Through Histone Methylation

In addition to their role in histone acetylation, ING proteins are critical regulators of histone methylation, an essential epigenetic mechanism involved in gene expression control. Histone methylation occurs through the addition of methyl groups to lysine or arginine residues on histones, catalyzed by histone methyltransferases (HMTs) [49,50]. Unlike histone acetylation, which generally promotes gene activation, the effects of histone methylation depend on the specific residue and degree of methylation. For instance, H3K4me1 typically inhibits gene transcription and defines chromatin “boundaries”, limiting the recruitment of “reader” proteins that interact with H3K4me3, thereby preventing their spread across these boundaries [51].

ING family proteins regulate gene expression in various neurobiological processes by recognizing specific histone methylation marks and recruiting regulatory factors, such as modifying enzymes. The PHD fingers of ING proteins possess sophisticated histone-sequence-reading capabilities, which are modulated by the interactions between different histone modifications [52]. These PHD fingers read the N-terminal tail of histone H3, mainly recognizing methylation markers on H3K4 [53], particularly H3K4me2 and H3K4me3. By recognizing these marks, ING proteins recruit multiprotein complexes, chromatin regulators, and transcription factors that regulate gene expression. For example, ING1 promotes active DNA demethylation at H3K4me3 by recruiting the DNA damage response factor Gadd45α [54]. Disruptions in this mechanism can lead to the hypermethylation of critical gene promoters, such as the retinoic acid-related orphan receptor alpha (*RORA*), which has been implicated in neurodevelopmental disorders like autism spectrum disorder (ASD) [55,56].

Similarly, studies have shown that ING2 promotes alternations in gene expression by binding to H3K4me3 and modulating chromatin modifications, which is crucial for early embryonic development. The downregulation of ING2 during mouse embryonic development results in developmental arrest, underscoring its critical role in regulating development. This occurs through ING2’s recruitment of HDAC to the *P21* promoter region, promoting acetylation and enhancing P21 transcription (Figure 2) [57]. In human fetal development, ING2 also suppresses the transcription of the *γ-globin* gene by recognizing H3K4me3 and recruiting the HDAC complex, which reduces acetylation at this site [58]. Moreover, ING5 participates in acetyltransferase complexes such as MOZ/MORF and HBO1, co-regulating embryonic stem cell self-renewal and differentiation in conjunction with H3K4 methylation catalyzed by Set1A [59].

In summary, ING proteins serve as integral regulators of histone methylation by recognizing methylation marks and orchestrating the recruitment of chromatin-modifying complexes. Through these mechanisms, they play a crucial role in developmental processes, cellular differentiation, and neurobiological function. The dysregulation of ING-protein-mediated histone methylation is increasingly recognized as a contributing factor in neurodevelopmental and neurodegenerative disorders, highlighting their potential as therapeutic targets in epigenetic-based interventions.

### 3.3. The Role of ING Proteins in DNA Methylation and DNA Damage Repair

ING proteins are crucial for maintaining genomic integrity in neurobiology, where they influence both DNA modification and DNA damage repair [4,10]. By coordinating processes such as DNA methylation and DNA repair pathways, ING proteins help regulate gene expression and protect neurons—highly specialized cells that are particularly susceptible to genomic insults [60,61].

A key aspect of this regulation involves the interaction of ING proteins with DNA methyltransferases (DNMTs) and demethylases, thereby modulating DNA methylation patterns. For example, ING1 binds H3K4me3 and recruits the growth-arrest and DNA damage protein Gadd45a, facilitating gene-specific DNA demethylation through base excision repair [62]. The loss of ING1 function can undermine this process, emphasizing its importance in maintaining appropriate methylation levels. Additionally, ING2 is involved in DNA methylation and demethylation, contributing to the dynamic regulation of gene expression.

**Figure 3:**
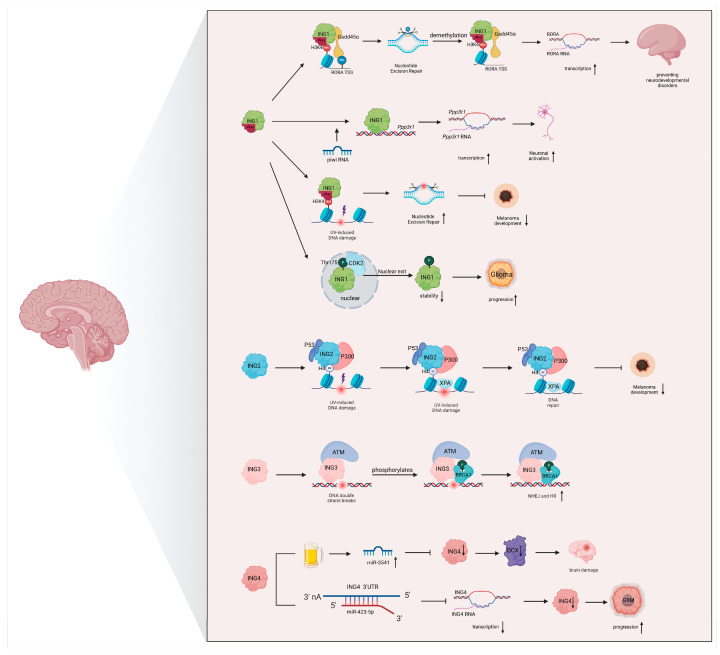
ING proteins in nucleic acid regulation and other modifications. Schematic representation of the regulatory mechanisms of ING proteins in the nervous system through interactions with DNA methylation, DNA damage repair, non-coding RNAs, and other epigenetic modifications. Abbreviations: RORA, retinoic acid-related orphan receptor alpha; Ppp3r1, protein phosphatase 3 regulatory subunit B, alpha; CDK2, cyclin dependent kinase 2; P53, p53 orthologs from arthropods; P300, E1A binding protein p300; XPA, DNA damage recognition and repair factor; ATM, ATM serine/threonine kinase; BRCA1, BRCA1 DNA repair associated; DCX, doublecortin. This graph was created with BioRender.com.

Beyond DNA methylation, ING proteins are integral to multiple DNA damage repair pathways. ING proteins can mediate the DNA damage repair process. ING1, in particular, has emerged as a key factor in coordinating DNA repair and apoptosis by binding to H3K4me3 via its PHD domain. This interaction is essential for nucleotide excision repair (NER) in responding to UV-induced DNA damage, underscoring the importance of its normal function in the DNA damage response. By contrast, mutated ING1 cannot effectively promote DNA repair (Figure 3) [63]. ING2, on the other hand, cooperates with tumor suppressor protein p53 and acetyltransferase p300 to promote histone H4 acetylation and chromatin relaxation. This modification facilitates the recruitment of DNA repair proteins, such as XPA (xeroderma pigmentosum group A protein), to DNA damage sites, thereby enhancing the repair efficiency of UV-induced damage [21,64]. This process is especially critical for the early recognition and repair of DNA damage in neurons and other highly active cell types. In addition to these roles, ING3 is involved in the repair of DNA double-strand breaks. ING3 recruits ATM kinase to the damage site, where it is activated and phosphorylates downstream targets like *BRCA1*. This activation promotes the recruitment and activity of downstream DNA damage repair proteins, facilitating both non-homologous end joining (NHEJ) and homologous recombination (HR) repair pathways [41,65,66], which are essential for maintaining genomic integrity in response to double-strand breaks.

In summary, ING proteins play important roles in DNA modification and DNA damage repair through interactions with key factors involved in histone modification, DNA methylation, and the repair pathway. Their involvement in these processes is essential for maintaining genome stability, regulating gene expression, and supporting the repair of DNA damage, particularly in highly specialized cells such as neurons.

### 3.4. Non-Coding RNAs and Their Interaction with ING Proteins

Non-coding RNAs (ncRNAs), including long non-coding RNAs (lncRNAs), microRNAs (miRNAs), Piwi-interacting RNAs (piRNAs), and others, are RNA molecules that do not encode proteins [67]. These ncRNAs, despite lacking the capacity to encode proteins, exert significant regulatory effects by interacting with RNAs (mRNAs or ncRNAs), DNA, and proteins [9]. These interactions significantly influence gene expression and are indispensable for various processes such as neuronal development, differentiation, and functional maintenance within the nervous system [13].

Emerging evidence suggests that ING proteins engage in complex interactions with ncRNAs, integrating these regulatory molecules into epigenetic control mechanisms. For example, doublecortin (DCX), a marker of neurogenesis that stabilizes microtubules, is significantly downregulated in the hippocampus following chronic heavy alcohol consumption. This effect may be mediated through miR-3541, which inhibits ING4 expression, leading to disrupted neurogenesis [68,69]. Similarly, ING proteins have been found to interact with ncRNAs to modulate neuronal gene expression in an activity-dependent manner. Specifically, piRNAs play a crucial role in ING1 recruitment to the *Ppp3r1* upstream regulatory element (URE), thereby enhancing *Ppp3r1* transcription in response to neuronal activation, which is essential for synaptic function and plasticity (Figure 3) [4].

Beyond their roles in neuronal physiology, ING proteins are also implicated in neural tumorigenesis through their regulation by miRNAs. For instance, miR-423-5p functions as an oncogene by directly targeting the 3′-UTR of *ING4*, thereby suppressing its expression and promoting glioma progression [70]. This suggests that ING proteins not only interact with ncRNAs but also serve as downstream targets of ncRNA-mediated gene regulation, further integrating them into the broader epigenetic landscape of neurobiology.

Overall, the interactions between ING family proteins and ncRNAs form a complex and intricate part of the epigenetic regulatory network. Interestingly, whether ING proteins, as part of the HDAC complex, regulate the transcription and regulation of ncRNA remains an unresolved question.

### 3.5. ING Proteins in Other Epigenetic Modifications

ING proteins participate in various epigenetic modifications beyond histone regulation, including post-translational modifications (PTMs) such as SUMOylation, ubiquitination, and phosphorylation. These modifications influence key cellular processes such as cell cycle regulation, apoptosis, and stress responses. However, their functions in the nervous system remain largely unexplored. For instance, ING2 undergoes SUMOylation at lysine 195 by SUMO1, which enhances its binding affinity to the Sin3A complex. Once SUMOylated, ING2 associates with the promoter region of TMEM71, facilitating the recruitment of the Sin3A/HDAC complex to repress gene expression [71]. This indicates that SUMOylation is an essential regulatory mechanism controlling ING2’s transcriptional functions, potentially influencing neuronal differentiation and survival. Notably, ING1 possesses a polybasic region (PBR) and a recently identified ubiquitin-binding domain (UBD), which enables it to bind ubiquitin, thereby inhibiting the polyubiquitination of p53 and protecting it from proteasomal degradation [72]. Additionally, ING1 stabilizes p53 levels by interacting with the deubiquitinating enzyme HAUSP. This mechanism connects lipid stress signals to ubiquitin-mediated proteasomal degradation, highlighting the key role of ING1 in cellular stress responses and protein stability [72].

A study on gliomas revealed that cyclin-dependent kinase 2 (CDK2) physically interacts with ING1 and phosphorylates it at threonine 152. This phosphorylation event triggers the relocalization of ING1 from the nucleus to the cytoplasm, reducing its stability. Such nucleocytoplasmic relocalization decreases ING1 expression levels, thereby impairing its pro-apoptotic function (Figure 3) [73]. Given the importance of apoptosis and cell cycle control in neuronal development and neurodegenerative diseases, ING1 phosphorylation may also have implications for neuronal survival and plasticity.

In summary, ING family members regulate critical processes such as cell growth, apoptosis, and tumorigenesis through mechanisms involving ubiquitination, phosphorylates, and SUMOylation. These regulatory pathways complement ING’s well-established roles in histone modifications and provide novel insights into the diverse functions of ING proteins, particularly in neurobiology and neurodevelopment.

Finally, to more clearly illustrate the role and mechanistic relationships of ING proteins in the nervous system, we created Table 1 and Table 2 based on neurological disorders and mechanisms, respectively.

## 4. The Potential Role of ING Proteins in Neurological Disorders

As our understanding of ING protein functions continues to deepen, increasing evidence suggests that the mechanisms underlying the roles of ING proteins in neurodevelopment and neuro-oncology are not yet fully elucidated, and ING proteins may also play a pivotal role in the onset and progression of other neurological disorders. In neurodegenerative diseases such as Alzheimer’s disease (AD) and Parkinson’s disease (PD), apoptosis and cellular senescence are key pathological mechanisms [74,75]. ING proteins have been shown to regulate processes like apoptosis and cellular senescence through epigenetic pathways [76]. For example, the upregulation of ING5 in U87 cells leads to the inhibition of proliferation and induces cell cycle arrest at the G2/M phase [77]. This suggests that ING proteins may be involved in the onset and progression of neurodegenerative diseases by inhibiting neuronal apoptosis and senescence.

Additionally, ING1 interacts with 14-3-3 proteins, facilitating its binding with *BAX*, which increases mitochondrial membrane permeability and ultimately regulates cellular adaptability to stress responses [78]. Interestingly, stress responses are closely associated with various neurological disorders, such as stroke or cerebral ischemic injury [79,80]. Therefore, ING proteins may serve a neuroprotective function in these conditions.

As epigenetic regulators, ING proteins recognize histone methylation marks and recruit HATs or HDACs to regulate the transcription of nearby genes [7]. Notably, HDAC inhibition has been shown to alleviate repetitive behaviors and social interaction deficits associated with ASD, restore memory performance, and induce transcriptomic changes similar to those observed with the antipsychotic drug trifluoperazine [81,82], highlighting the critical role of HDAC in patients with ASD. In addition, mutations in the human PHD domain can lead to neurodevelopmental disorders, such as intellectual disability, global developmental delay, and expressive language impairment [83,84]. Furthermore, array comparative genomic hybridization (array CGH) analysis has identified mutations in ING2 in patients with intellectual disabilities [85]. These findings further indicate the potential role of ING proteins in disorders related to neural tube development, which warrants further investigation.

Notably, ING proteins are closely associated with the regulation of genes critical for nervous system development. These genes are involved in neuronal differentiation, synaptic plasticity, and neurodevelopment. Such functions are implicated in various neurological disorders, including Alzheimer’s disease, autism, and post-traumatic stress disorder (PTSD). Although current studies have not yet established a direct link between ING proteins and these disorders, their indirect involvement suggests that ING proteins hold significant potential in the context of neurological diseases.

## 5. Perspectives and Conclusion

By virtue of their unique PHD domains, ING proteins can recognize and bind to specific histone methylation marks, facilitating histone modifications that influence chromatin structure and function. This is critical in processes such as cell fate determination, neuro-oncogenesis, and nervous system development. For example, H3K4me3 recruits the ING2-HDAC complex to reduce acetylation levels at that site. However, different members of the ING protein family play distinct roles: ING3 and ING5 tend to interact with HAT complexes to promote gene transcription, while ING1, ING2, and ING4 are more inclined to recruit HDAC complexes to inhibit transcription. Dysregulated acetylation levels due to altered ING protein activity contribute significantly to the development of neurotumors and neurodevelopmental disorders.

In addition, ING1 plays a critical role in DNA demethylation, a process that involves the conversion of 5-hydroxymethylcytosine (5hmC) to 5-formylcytosine (5fC) and then to 5-carboxylcytosine (5caC) [86,87]. Notably, 5fC has been identified as dynamically distributed in activated neurons [88], while ING1 is known to regulate gene expression in such neurons [4]. Sequencing data suggest that ING1 may act as a potential reader of 5fC [89], indicating that its regulation of gene expression in activated neurons could occur through recognizing regions enriched in 5fC. Our previous studies have also shown that DNA demethylation undergoes redistribution in response to fear extinction learning [90]. We hypothesize that during fear extinction, ING1, as an epigenetic regulator, plays a pivotal role in activated neurons by recognizing the accumulation of 5fC.

The interaction between ING proteins and non-coding RNAs adds an additional layer of gene regulation. Their involvement in miRNA and piRNA pathways influences neuronal activation [4], further suggesting that these proteins could serve as potential therapeutic targets for psychiatric disorders such as PTSD. Spruijt, C. G. et al. demonstrated that the ING1 protein was pulled down using 5fC in mouse embryonic stem cells [89], further highlighting the important role of ING1 in developmental processes. ING1 may specifically recognize 5fC accumulation through an as-yet-unidentified domain, thereby recruiting HATs and other histone acetyltransferases to promote chromatin opening [7], which in turn enhances the transcription of differentiation-related genes.

During early embryonic development, neural progenitor cells (NPCs) undergo a tightly regulated process of differentiation into neurons, astrocytes, and oligodendrocytes. This transition is controlled by epigenetic mechanisms, including histone modifications and DNA methylation. ING proteins, particularly ING1 and ING5, have been shown to interact with histone-modifying complexes that regulate chromatin accessibility and lineage-specific gene activation. Studies have demonstrated that ING proteins act as chromatin readers, recognizing H3K4me3-enriched promoters, which are marks of transcriptionally active genes. Given that H3K4me3 is essential for lineage commitment, it is plausible that ING proteins contribute to neurogenic versus gliogenic fate decisions by recruiting chromatin-modifying enzymes to key developmental genes [91]. Whether ING proteins actively participate in the repression of alternate lineages (e.g., glial differentiation suppression during early neuronal development) remains an open question.

In conclusion, ING proteins play a pivotal but underappreciated role in neurodevelopment, particularly in chromatin remodeling, histone modifications, and neuronal gene regulation. While much is known about their function in tumor suppression, their contributions to neuronal differentiation, synaptic plasticity, and neurodevelopmental disorders remain largely unexplored. Investigating how ING proteins integrate into neurodevelopmental signaling pathways, regulate neural progenitor cell fate, and contribute to neuronal connectivity will be crucial for understanding their full biological significance. Future studies should also explore their therapeutic potential as epigenetic modulators, which may pave the way for novel interventions in neurodevelopmental and neurodegenerative diseases.

## Figures and Tables

**Table 1:** The roles and mechanisms of ING proteins in the nervous system (classification according to neurological disorders).

Phenotype	Animal Models or Clinical Trials	Role and Mechanism	References
Brain tumor	Mouse and clinical trial	By recruiting HDACs to regulate chromatin accessibility, it inhibits the transcription of cancer-related genes; by recruiting HATs to regulate chromatin accessibility, it promotes the transcription of cancer-related genes; it participates in DNA damage repair and undergoes nuclear export and degradation after self-phosphorylation, and its transcription is inhibited by miRNAs.	[6,35,36,40,41,42,43,44,45,58,59,60,61,65,68,73]
Neurodevelopment	Mouse	By recruiting HDACs to regulate chromatin accessibility, it suppresses the transcription of differentiation-related genes; by recruiting HATs to regulate chromatin accessibility, it promotes the transcription of differentiation-related genes.	[38,39,54,55,56]
Neurodevelopmental disorders	Mouse and clinical trial	By promoting promoter demethylation through the NER pathway, it facilitates the transcription of genes involved in preventing neurodevelopmental disorders.	[51,52,53]
Brain damage	Clinical trial	By promoting promoter demethylation through the NER pathway, its transcription is inhibited by miRNAs.	[63,64]
Neuronal activation	Mouse	It promotes the expression of genes related to neural function.	[4]

**Table 2:** The roles and mechanisms of ING proteins in the nervous system (classification according to mechanism).

Mechanism	Phenotype	References
Histone acetylation	Brain tumor; neurodevelopment; brain damage	[6,35,36,38,39,40,41,42,43,44,45]
Histone methylation	Brain tumor; neurodevelopment; neurodevelopmental disorders; brain damage; neuronal activation	[4,51,52,53,54,55,56,58,59]
DNA methylation and DNA damage repair	Brain tumor; neurodevelopment; brain damage; neuronal activation	[4,60,61,62,63,64,65]
Non-coding RNAs	Brain tumor; brain damage; neuronal activation	[63,68,69]
Other epigenetic modifications	Brain tumor	[73]

## Data Availability

Not applicable.

## Abbreviations

ING	Inhibitor of growth
NuRD	Nucleosome remodeling and deacetylation complex
HAT	Histone acetyltransferase
HDAC	Histone deacetylase
LID	Lamin-interacting domain
NLS	Nuclear localization signal
PHD	Plant homeodomain
LZL	Leucine zipper-like
PCNA	Proliferating cell nuclear antigen
PIP	PCNA-interacting protein
PBD	Partial bromodomain
PBR	Polybasic region
PIs	Phospholipids
Ub	Ubiquitin
TET	Ten-Eleven Translocation
TOPD	TET-OGT-PROSER1-DBHS
PD	Parkinson’s disease
ASD	Autism spectrum disorder
BTICs	Brain-Tumor-Initiating Cells
MOZ	Monocytic leukemia zinc finger protein
MORF	MOZ-related factor
FSH	Follicle-stimulating hormone
HMTs	Histone methyltransferases
RORA	Retinoic acid-related orphan receptor alpha
DNMTs	DNA methyltransferases
NER	Nucleotide excision repair
ncRNAs	Non-coding RNAs
miRNAs	MicroRNAs
lncRNAs	Long non-coding RNAs
UBD	Ubiquitin-binding domain
AD	Alzheimer’s disease
5hmC	5-hydroxymethylcytosine
5fC	5-formylcytosine
5caC	5-carboxylcytosine
